# Activation of angiotensin-converting enzyme 2 produces an antidepressant-like effect via MAS receptors in mice

**DOI:** 10.1186/s13041-023-01040-y

**Published:** 2023-06-13

**Authors:** Osamu Nakagawasai, Kohei Takahashi, Taisei Koyama, Ryota Yamagata, Wataru Nemoto, Koichi Tan-No

**Affiliations:** 1 Division of Pharmacology, Faculty of Pharmaceutical Sciences, Tohoku Medical and Pharmaceutical University, 4-4-1 Komatsushima, Aoba-ku, Sendai, 981-8558 Miyagi Japan; 2 Department of Pharmacology, School of Pharmacy, International University of Health and Welfare, 2600-1 Kitakanemaru, Ohtawara, 324-8501 Tochigi Japan

**Keywords:** Antidepressant, Angiotensin-converting enzyme 2, Ang (1–7), Diminazene, MAS receptor

## Abstract

Angiotensin (Ang)-converting-enzyme (ACE) 2 converts Ang II into Ang (1–7), which in turn acts on MAS receptors (ACE2/Ang (1–7)/MAS receptors pathway). This pathway has neuroprotective properties, making it a potential therapeutic target for psychiatric disorders such as depression. Thus, we examined the effects of diminazene aceturate (DIZE), an ACE2 activator, on depressive-like behavior using behavioral, pharmacological, and biochemical assays. To determine whether DIZE or Ang (1–7) produce antidepressant-like effects, we measured the duration of immobility of mice in the tail suspension test following their intracerebroventricular administration. Next, we measured the levels of ACE2 activation in the cerebral cortex, prefrontal cortex, hippocampus, and amygdala after DIZE injection, and examined which cell types, including neurons, microglia, and astrocytes, express ACE2 in the hippocampus using immunofluorescence. Administration of DIZE or Ang (1–7) significantly shortened the duration of immobility time in the tail suspension test, while this effect was inhibited by the co-administration of the MAS receptor antagonist A779. DIZE activated ACE2 in the hippocampus. ACE2 was localized to neurons, astrocytes, and microglia in the hippocampus. In conclusion, these results suggest that DIZE may act on ACE2-positive cells in the hippocampus where it increases the activity of ACE2, thereby enhancing signaling of the ACE2/Ang (1–7)/MAS receptor pathway and resulting in antidepressant-like effects.

**Supplementary Information:**

The online version contains supplementary material available at 10.1186/s13041-023-01040-y.

## Introduction

The number of depressed patients in the world is expected to rise due to the impact of the COVID-19 pandemic [1–3]. Based on the monoamine hypothesis, which proposes a biochemical explanation for depression, drugs that increase synaptic levels of monoamines such as serotonin (5-HT) and noradrenalin have been used in the treatment of depression. Currently, selective serotonin reuptake inhibitors and serotonin noradrenalin reuptake inhibitors are the mainstay of drug therapy. However, it is estimated that about 30% of depressed patients do not improve when treated with current antidepressants [4]. It is clear that further elucidation of the mechanisms underlying the onset of depression is needed, as well as the development of new drugs and therapies.

The Renin-angiotensin (Ang) system (RAS) is a regulatory system for cardiovascular functions such as blood pressure, water and electrolyte balance [5, 6]. In the RAS, angiotensinogen (AGT) is metabolized to Ang I by renin, and Ang I is converted to Ang II by angiotensin-converting-enzyme (ACE). Ang II is the major active peptide of the RAS, and is involved in vasoconstriction [7], inflammatory responses [8], oxidative stress [9], and vasoconstriction via the Ang II Type 1 (AT1) receptor, a G protein-coupled receptor. In contrast, AT2 receptors play a protective role against the RAS [10, 11]. It has been shown that the above-mentioned hyperfunction of the ACE/Ang II/AT1 receptor system is involved in depression as well as in the cardiovascular system [12]. For example, administration of captopril, an ACE inhibitor, to depressed patients with concurrent hypertension improved their depressive symptoms [13, 14]. In parallel, the RAS has an intrinsic mechanism that counterbalances the ACE/Ang II/AT1 receptor system, and is referred to the ACE2/Ang (1–7)/MAS receptor system [15, 16]. Ang (1–7) is an N-terminal fragment of Ang II generated by ACE2 [17] that antagonizes the ACE/Ang II/AT1 receptor system by exerting physiological effects such as vasodilation [18, 19], anti-inflammatory effects [16], and antioxidant effects [20] via MAS receptors. Therefore, the inhibition of the ACE/Ang II/AT1 receptor system could potentially represent a novel therapeutic target for depression [21].

Diminazene aceturate (DIZE) is an aromatic diamidine approved by the U.S. Food and Drug Administration, and has been used in veterinary medicine for over 60 years to treat trypanosomiasis and babesiosis. Recent studies have reported that DIZE enhances the catalytic activity of ACE2 by direct binding [22]. Indeed, the administration of DIZE in a diabetic mouse model activated ACE2 and increased Ang (1–7) production [23]. Furthermore, the intracerebroventricular (i.c.v.) administration of Ang (1–7) has been observed to shorten immobility time in the forced swimming test [24]. Thus, DIZE activation of the ACE2/Ang (1–7)/MAS receptor system in the brain may represent an effective therapeutic strategy for depression.

In the present study, to investigate whether ACE2 activation in the brain could be a novel therapeutic target for depression, we examined the effects of DIZE on the duration of immobile behavior using a behavioral-pharmacological test, and explored some mechanisms of action using biochemical and histological assays.

## Results

### DIZE shows antidepressant-like effect in the tail suspension test

**Fig. 1:**
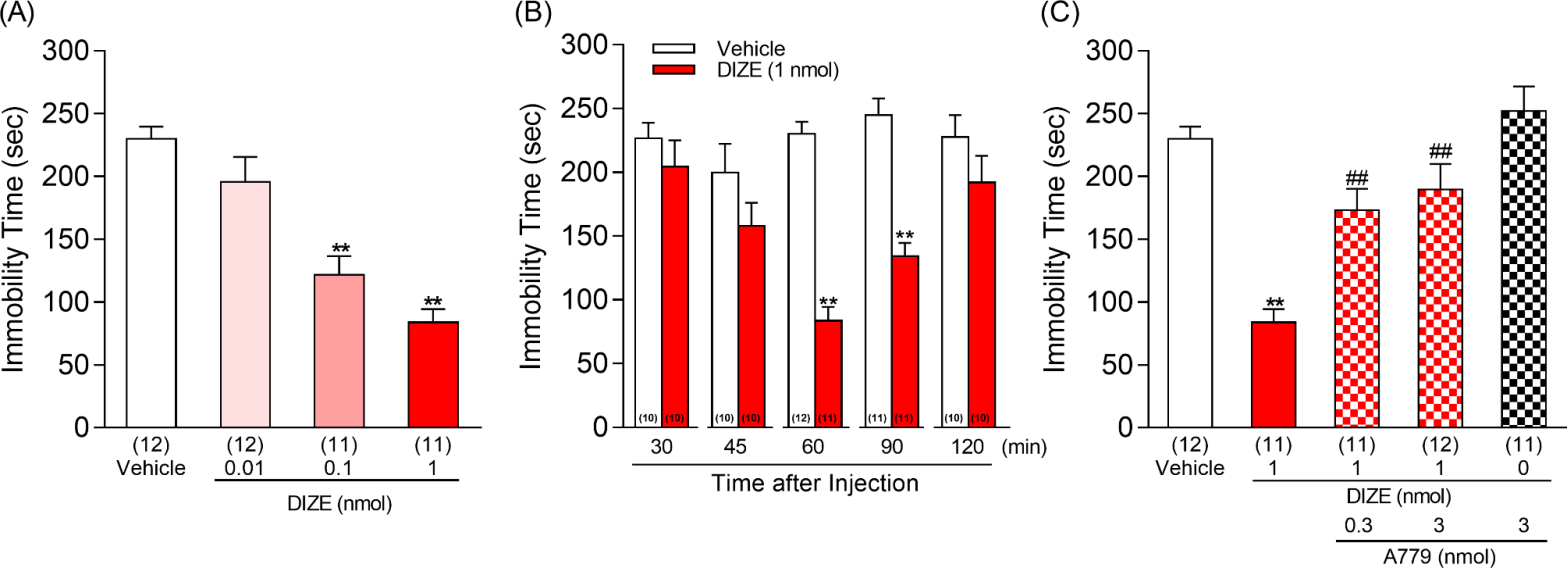
Effects of DIZE on the tail suspension test in mice. **A:** The immobility time was measured 60 min after the administration of vehicle or DIZE (0.01, 0.1, 1 nmol). **B:** Time courses of the DIZE (1 nmol)-induced antidepressant-like effect in mice. **C:** Effects of A779 on the DIZE-induced reduction in immobility time in mice. Vehicle, DIZE (1 nmol) or DIZE (1 nmol) in combination with A779 (0.3 or 3 nmol) were administered 60 min prior to measurements. One-way ANOVA: F (3, 42) = 21.24, p < 0.0001, Fig. 1**(A)**, F (4, 52) = 15.96, p < 0.0001, Fig. 1**(C)**. Two-way ANOVA: [time: F (4, 95) = 4.671, p = 0.0017; treatment: F (1, 95) = 51.07, p < 0.0001; time × treatment: F (4, 95) = 6.079, p = 0.0002, Fig. 1**(B)**]. Values represent the means ± SEM. Numbers in parentheses indicate the number of animals in each group. **p < 0.01 compared with vehicle-treated mice. ##p < 0.01 compared with DIZE (1 nmol)-treated mice

To determine whether DIZE, an ACE2 activator, exerts antidepressant effects, mice were administered DIZE i.c.v., and a tail suspension test was performed 60 min after administration. DIZE dose-dependently reduced immobility time in mice (Fig. 1A). This decrease in immobility time or anti-depressant-like effects was observed not only after 60 min but also 90 min following DIZE administration at a dose of 1nmol (Fig. 1B). The immobility time in the tail suspension test is influenced by locomotor activity. In other words, if locomotor activity is enhanced by a drug treatment then the immobility time may be shortened and lead to a misinterpretation of the drug’s effect on despair. Thus, we measured the change in spontaneous locomotor activity of mice treated with DIZE. No significant difference was observed between the vehicle- and DIZE-treated groups in locomotor activity at any time points (Supplementary Fig. S1A). The total locomotor activity of the mice calculated for the 90 min period also showed no significant difference between the vehicle and DIZE groups (Supplementary Fig. S1B). These results indicated that the reduction of the immobility time induced by DIZE (1 nmol) was not due to changes in spontaneous locomotor activity.

### A779 abolishes DIZE-induced antidepressant-like effect in mice

By activating ACE2, DIZE may produce it’s antidepressant-like effects as a result of an increase in Ang (1–7) acting on MAS receptors. We tested this hypothesis using A779, an antagonist of the MAS receptor on which Ang (1–7) acts. The antidepressant-like effect of DIZE (1 nmol) was found to be completely abolished by the co-administration of A779 (0.3, 3 nmol) (Fig. 1C). These results indicated that the MAS receptor may be involved in the antidepressant-like effects of DIZE.

## Ang (1–7) shows antidepressant-like effect in the tail suspension test

**Fig. 2:**
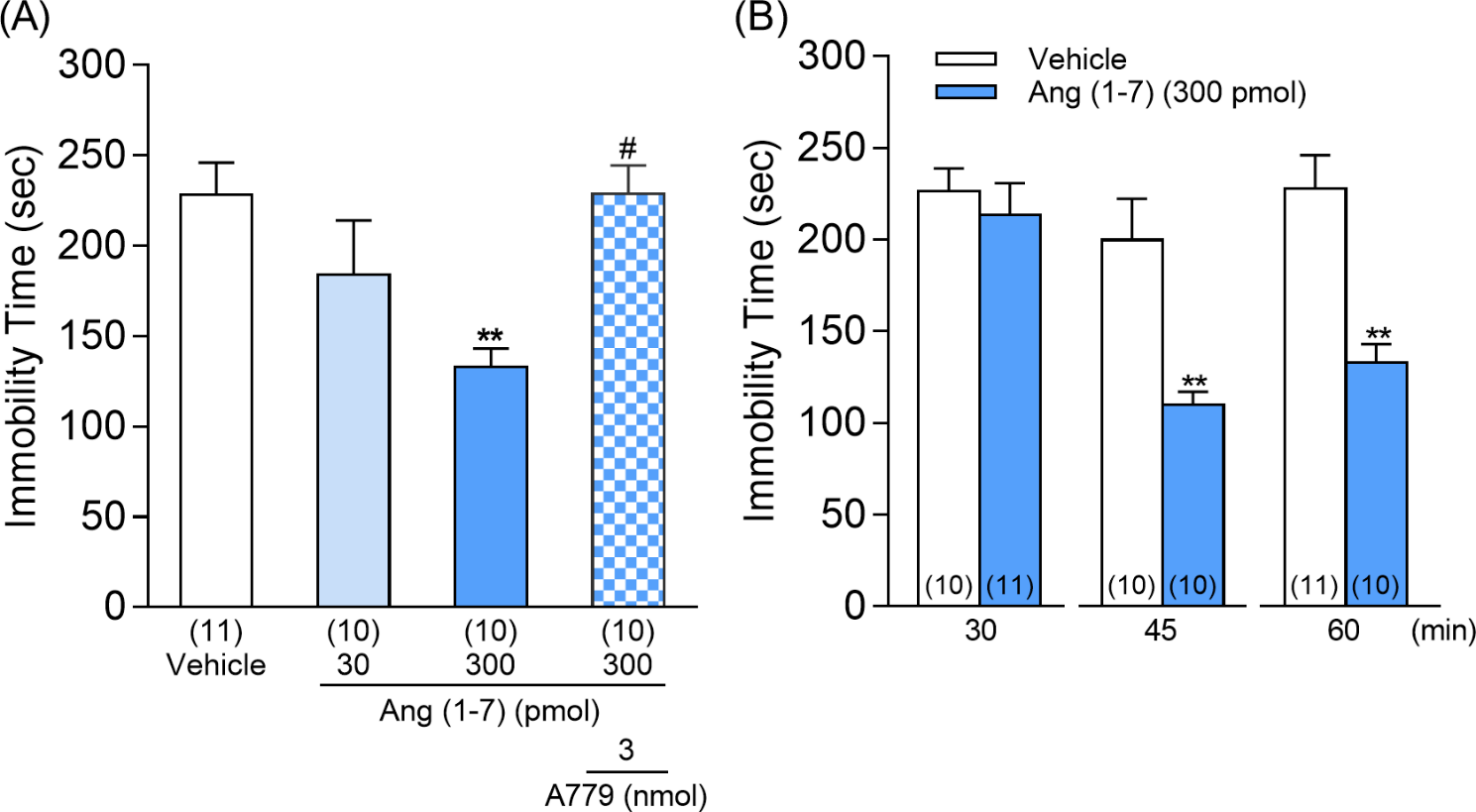
Effects of Ang (1–7) on the tail suspension test in mice. **A:** The immobility time was measured 60 min after the administration of vehicle, Ang (1–7) (30 or 300 pmol) or Ang (1–7) (300 pmol) in combination with A779 (3 nmol). **B:** Time courses of the Ang (1–7) (300 pmol)-induced antidepressant-like effect in mice. One-way ANOVA: F (3, 37) = 5.265, p = 0.0040, Fig. 2**(A)**. Two-way ANOVA: [time: F (2, 56) = 8.822, p = 0.0005; treatment: F (1, 56) = 26.82, p < 0.0001; time × treatment: F (2, 56) = 4.369, p = 0.0172, Fig. 2**(B)**]. Values represent the means ± SEM. Numbers in parentheses indicate the number of animals in each group. **p < 0.01 compared with vehicle-treated mice. #p < 0.05 compared with Ang (1–7) (300 pmol)-treated mice

The antidepressant-like effects of DIZE were found to involve MAS receptors, indicating that Ang (1–7) may mediate these effects. Therefore, to clarify whether Ang (1–7) exhibits antidepressant-like effects, Ang (1–7) was administered i.c.v. at different doses, and the tail suspension test was performed 60 min after administration. Ang (1–7) (300 pmol) administration significantly decreased immobility time in mice (Fig. 2A). This decrease was inhibited by a co-administration with A779 (3 nmol) (Fig. 2A). Furthermore, Ang (1–7) (300 pmol) showed antidepressant-like effects as soon as 45 and 60 min after i.c.v. administration (Fig. 2B). These results indicated that the antidepressant-like effects of DIZE are mediated through the MAS receptor by Ang (1–7), which is increased by ACE2 activation.

### Changes in brain ACE2 activity after DIZE administration

**Fig. 3:**
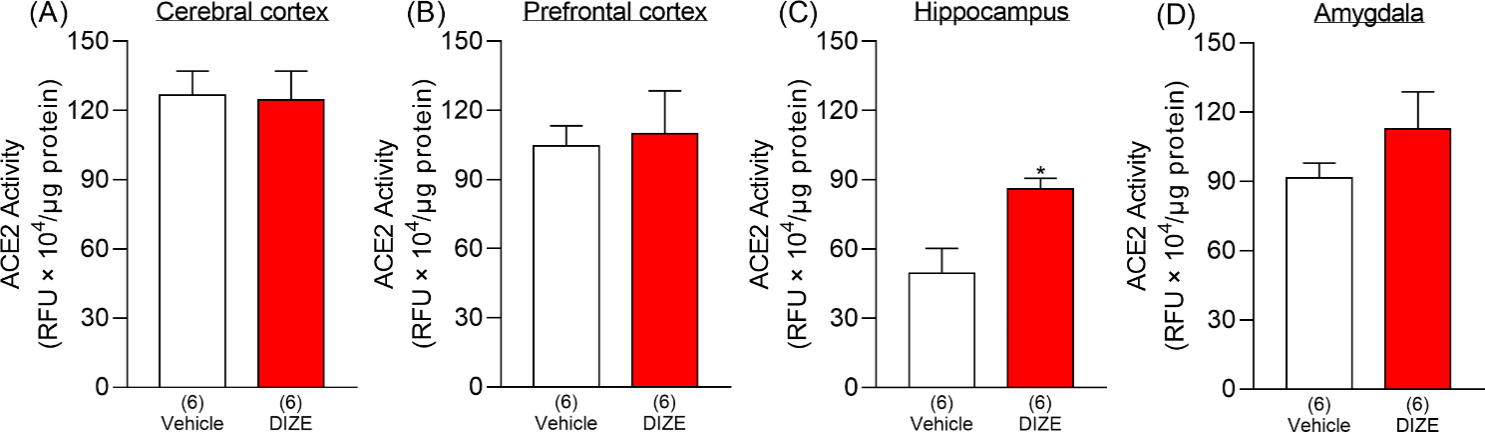
Changes in ACE2 activity in the brain of naïve mice following the i.c.v. administration of DIZE. Sixty min after the administration, samples of the cerebral cortex, prefrontal cortex, hippocampus, and amygdala were collected from the brain and homogenized to determine ACE2 activity. Student’s t-test: t = 0.1171, df = 10, p = 0.9091, Fig. 3**(A)**; t = 0.2676, df = 10, p = 0.7945, Fig. 3**(B)**; t = 3.137, df = 10, p = 0.0106, Fig. 3**(C)**; t = 1.22, df = 10, p = 0.2505, Fig. 3**(D)**. Values represent the means ± SEM. Numbers in parentheses indicate the number of animals in each group. *p < 0.05 compared with vehicle-treated mice

MAS receptor expression is known to be abundant in the hippocampus, cortex, and amygdala in both humans and mice (https://www.proteinatlas.org/ENSG00000130368-MAS1/brain). To determine in which of these brain regions DIZE activation of ACE2 occurs, brains were removed 60 min after DIZE (1 nmol) was administered i.c.v. then divided into 4 regions known to be involved in depression (hippocampus, cerebral cortex, amygdala, and prefrontal cortex). The treatment with DIZE significantly increased ACE2 activity in the hippocampus, while it did not affect the activity in other regions (Fig. 3).

### Localization of ACE2 in the hippocampus of naïve mice

**Fig. 4:**
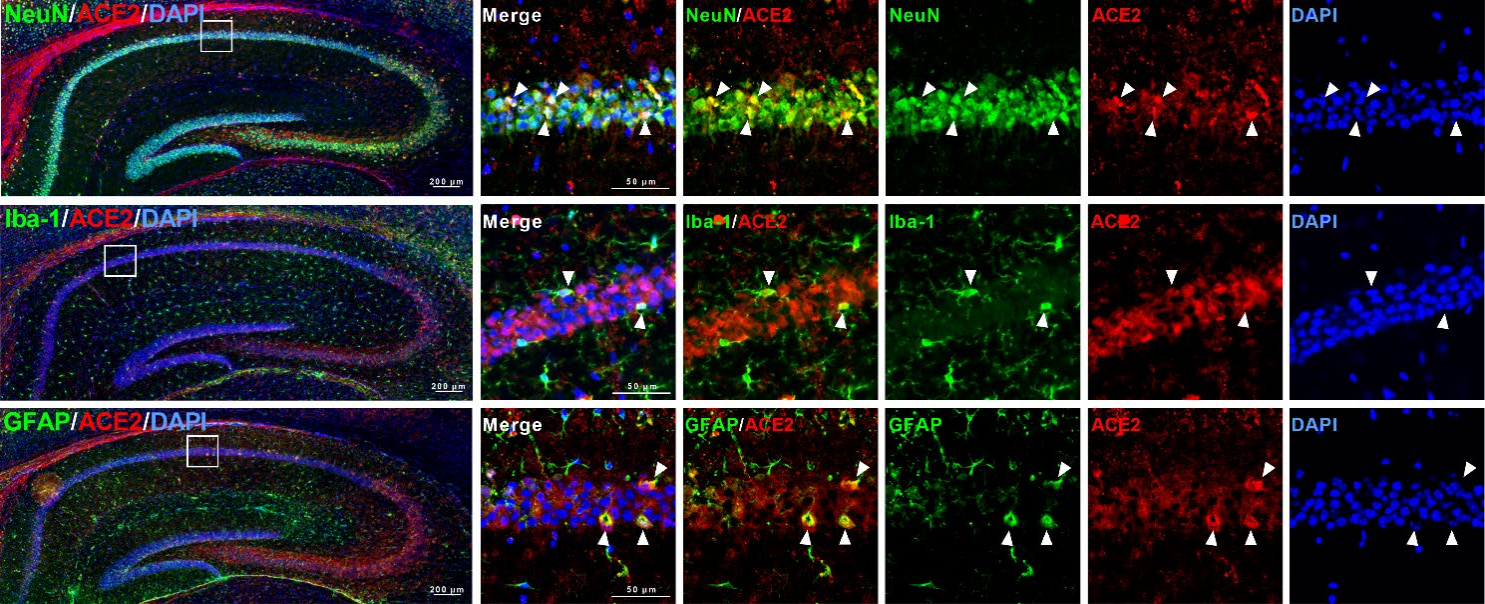
Double immunofluorescence staining for ACE2 and cell specific markers for neuron (NeuN), microglia (Iba-1) or astrocyte (GFAP) in the hippocampus of naïve mice. Photomicrographs showing fluorescent labeling for ACE2 (red), NeuN (green), Iba-1 (green), GFAP (green) or nuclei with DAPI (blue) in the hippocampus of naïve mice

As DIZE increased ACE2 activity in the hippocampus of mice, we examined which cell types, whether neurons, microglia, or astrocytes, express ACE2 in the hippocampus. ACE2 labeling was found in NeuN-, GFAP-, and Iba1-positive cells in the hippocampal cornu ammonis (CA) 1 (Fig. 4).

## Discussion

In this study, we showed that the ACE2 activator DIZE exhibited an antidepressant-like effect in mice through activation of MAS receptors via Ang (1–7).

DIZE has been found to alter the conformation of ACE2 by targeting and directly binding to a specific structural pocket in the hinge-bending region of ACE2, thereby enhancing its catalytic activity [22] and the conversion of Ang II into Ang (1–7) [23]. Earlier findings in rats suggested that Ang (1–7) has antidepressant-like effects. For example, when Ang (1–7) was administered i.c.v. to TGR (ASrAOGEN) 680 rats, it reduced their brain AGT levels and depression-like phenotype as they exhibited a decreased immobility time in a forced swimming test [24]. The administration of Ang (1–7) was also shown to improve the anxiety- and depressive-like behavior of hypertensive transgenic (mRen2)27 rats [25]. Moreover, the ACE2/Ang (1–7)/MAS receptors pathway, which is activated by exercise, has also been reported to improve cardiovascular and mental status [26]. From these findings, we hypothesize that the activation of the ACE2/Ang (1–7)/MAS receptor pathway in the central nervous system may induce antidepressant effect. In the present study, we found that DIZE administration shortened the duration of the immobile behavior in mice in the tail suspension test (Fig. 1A), but did not affect their locomotion when compared to the vehicle-treated group (Supplementary Fig. S1). DIZE produced a significant effect 60–90 min after its administration (Fig. 1B) which led us to consider that DIZE has antidepressant-like properties in mice. Next, we investigated whether the DIZE-induced reduction in immobility time was mediated by the MAS receptors on which Ang (1–7) acts. We found that the co-administration of DIZE and A779, a MAS receptor blocker, significantly inhibited the antidepressant-like effect of DIZE (Fig. 1C). These results suggest that DIZE exerts its antidepressant-like effects by increasing the production of Ang (1–7) which in turn activates MAS receptor signaling. Moreover, we examined whether Ang (1–7), which is produced by the activation of ACE2, can affect the immobility time in the tail suspension test. We found that compared to the vehicle-treated group, the immobility time was significantly decreased in mice treated with Ang (1–7), an effect that could be inhibited by the co-administration of the MAS receptor antagonist A779 (Fig. 2A). Taken together, these results suggest that the antidepressant-like effect of DIZE may have resulted from the activation of MAS receptors by Ang (1–7), which increased as a result of ACE2 activation.

**Fig. 5:**
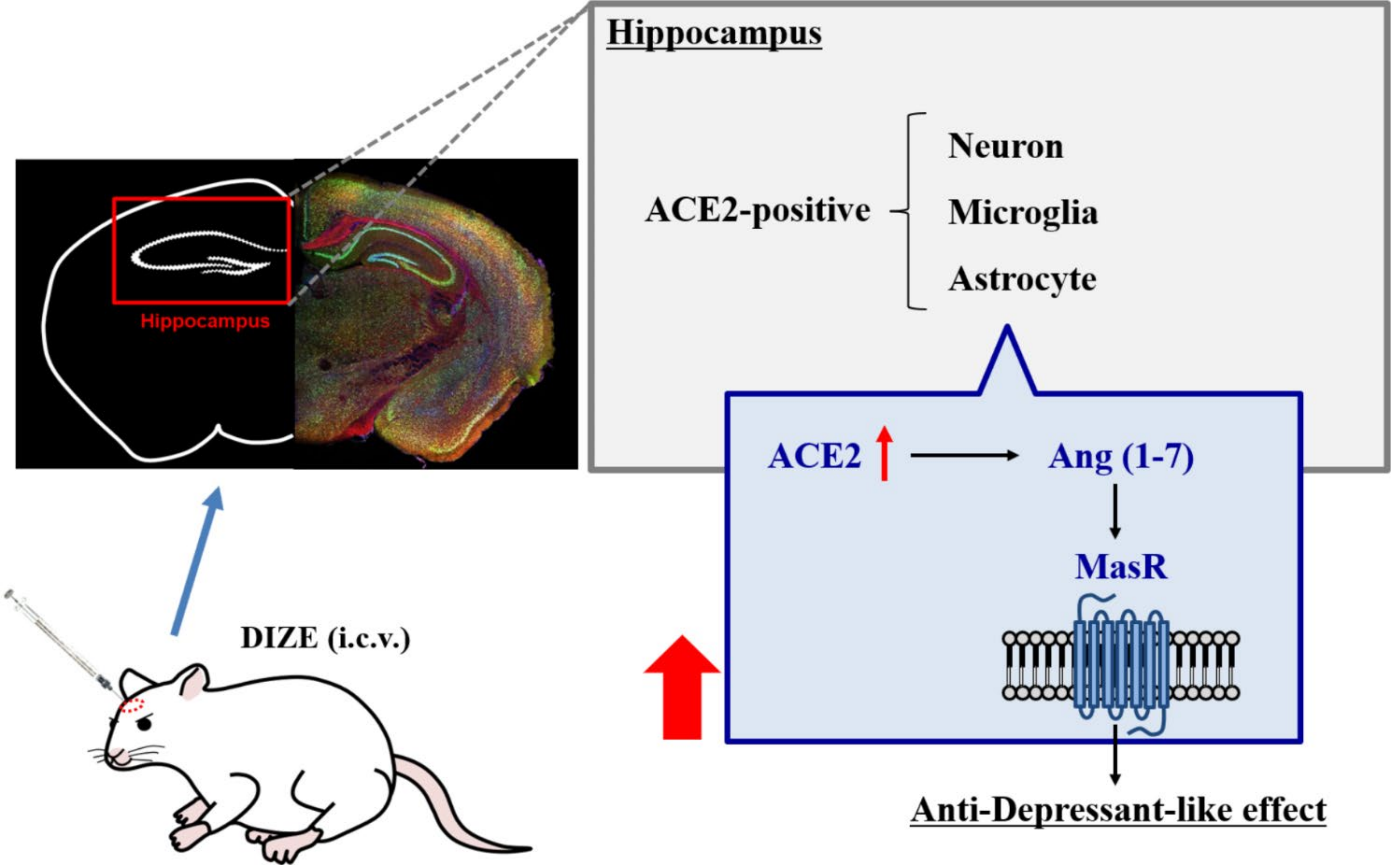
Hypothesis for DIZE antidepressant mechanism

We then measured ACE2 activity in brain regions implicated in depression (hippocampus, cerebral cortex, prefrontal cortex, and amygdala) to determine those that show ACE2 activation after i.c.v. administration of DIZE. We found a marked increase in ACE2 activity in the hippocampus (Fig. 3C). We next identified the cell types in the hippocampus that showed expression of ACE2 by immunohistochemical staining. Neurons, astrocytes and microglia showed ACE2 expression in the hippocampal CA1 (Fig. 4). Moreover, similar co-localization results were observed in other hippocampal regions (CA2, CA3, and dentate gyrus) (Supplementary Fig. S2). A study using TGR (ASrAOGEN) 680 rats, which exhibit reduced levels of AGT in the brain and an anxiety-like or depression-like phenotype, showed that 5-HT and its metabolite 5-HIAA were reduced in brain regions that control emotion, such as the hippocampus and prefrontal cortex [27]. Other research groups have reported that when the Ang II precursor Ang (1–12) was administered intraperitoneally to rats, plasma Ang II and Ang (1–7) were markedly elevated 5 min after [28]. This report indicates that the conversion of Ang II to Ang (1–7) by ACE2 activation takes place in a very short time. Here, the antidepressant-like effect of Ang (1–7) was not observed immediately after its administration but 45 min later whereas the reduction in immobility time was similar to that observed 60 min after administration (Fig. 2B). Although in our previous studies we have confirmed that MAS receptors are localized to neurons and microglia of the spinal cord [29] another study has demonstrated that, while the depletion of ACE2 reduced serotonin levels in the brain, ACE2 activation is required for running-induced hippocampal neurogenesis [30]. Hippocampal neurogenesis and serotonin are known to play an important role in antidepressant effects [31, 32]. These findings suggest that the activation of MAS receptors may have mediated antidepressant-like effects as a result of the modulation of other neuronal systems such as serotonergic pathways. Together with the results of the previous report, the present study suggests that ACE2 activity in ACE2-positive cells of the hippocampus enhances the ACE2/Ang (1–7)/MAS receptor system, leading to antidepressant effects (Fig. 5).

The present study revealed that DIZE may have antidepressant-like effects in naïve mice. It has been reported that the activation of ACE2 increases alamandine [33], an Ang (1–7)-like protein which, interestingly and similar to Ang (1–7), has been reported to exhibit antidepressant effects via Mas receptors [34]. Therefore, we plan to continue our investigation on the antidepressant effects of DIZE by quantifying the levels of Ang (1–7) and alamandine to confirm whether Ang (1–7) or alamandine actually increase in the brain after DIZE administration, and by performing acute and chronic administration of DIZE using animal models of depression such as olfactory bulbectomy, social defeat, or chronic mild stress.

In conclusion, our results suggest that intracerebroventricular administration of DIZE reduces immobility time in mice via activation of MAS receptors by Ang (1–7), which is increased by ACE2 activation, suggesting the potential use of ACE2 activators or MAS receptor agonists for antidepressant therapy. We hope to clarify the possibility that the activation of ACE2 in the brain may be a useful target for the development of new treatments for depression.

## Materials and methods

All experiments were performed in accordance with the Guide for the Care and Use of Laboratory Animals from the Tohoku Medical and Pharmaceutical University (Approval number: 20051-cn, 21035-cn) and the National Institutes of Health Guide for the Care and Use of Laboratory Animals. Efforts were made to minimize suffering and to reduce the number of animals used.

### Animals

Male ddY mice (weighing 28–32 g; Japan SLC, Shizuoka, Japan) were used in all experiments (total: n = 345, behavioral test: n = 331, ACE2 assay: n = 12, immunohistochemical study: n = 2). The mice were housed in cages with free access to food and water under conditions of controlled temperature (22 ± 2 °C) and humidity (55 ± 5%) on a 12-h light-dark cycle (lights on: 07:00 to 19:00).

### Drugs

DIZE (LKT Laboratories, Minnesota, USA), A779 (Bachem, Bubendorf, Switzerland), and Ang (1–7) (Peptide Institute, Osaka, Japan) were dissolved in Ringer’s solution. The compounds were administered i.c.v. alone or in combination to mice under diethyl ether inhalation anesthesia, at a volume of 5 µL using a 50 µL Hamilton microsyringe attached to a disposable 27-G needle. The dose for DIZE was obtained from a previous report [35].

### Tail suspension test

The tail suspension test was performed as previously described [36–38], to evaluate the antidepressant-like effects of DIZE or Ang (1–7). Mice were suspended with their tails taped, such that they were 30 cm above the floor. An investigator blinded to the treatment assignments observed the immobility time for 10 min.

### Locomotor activity

Locomotor activity was determined using the SUPERMEX monitoring system (Muromachi Kikai Co., Tokyo, Japan). The details of the apparatus have been previously described [36, 39, 40]. Briefly, the Supermex instrument can monitor minute movements in all three planes of motion (sagittal, coronal, and horizontal) as one movement owing to its infrared sensor with multiple Fresnel lenses, which can be moved close enough to the cage to capture multidirectional locomotor alterations in a single mouse. The Supermex instrument was connected to a behavioral analysis system (CompACT AMS, Muromachi Kikai) that can interpret each movement as one count. Locomotor activity was measured for 90 min during the light phase, between 11:00 am and 15:00 pm. Each mouse was placed in an activity box of SUPERMEX for 15 min for adaptation prior to injection with either vehicle or DIZE.

### Measurement of ACE2 activity

ACE2 activity was measured using the SensoLyte 390 ACE2 activity assay kit (AnaSpec, CA, USA) according to the supplied manual. Mice were injected i.c.v. with vehicle or DIZE, and brains were removed 1 h later. Brain slices were prepared in the following manner. The excised brains were sectioned into the hippocampus, cerebral cortex, prefrontal cortex, and amygdala using a brain slicer (Muromachi Kikai) and thoroughly homogenized in 150 µL of assay buffer (Component C buffer) provided with the kit. Homogenized samples were incubated for 15 min at 4 °C. After centrifugation (20,000 × g, 10 min, 4 °C), 120 µL of the supernatant was collected and used as samples. The brain samples were reacted with ACE2 substrate for 30 min under light-shielded conditions at room temperature, and after addition of reaction stopper solution, ACE2 activity was measured by measuring fluorescence intensity (excitation: 330 nm /fluorescence: 390 nm) on a SoftMax Pro (Molecular Devices, CA, USA). Total protein concentration was quantified by the Bradford protein assay and used to normalize ACE2 activity.

### Immunofluorescence study

Brain samples were collected as previously described [41, 42]. Mice were anesthetized by intraperitoneal administration of a combined triadic anesthesia: medetomidine (50 mg/kg; Nippon Zenyaku Kogyo Co., Ltd., Fukushima, Japan), midazolam (4 mg/kg; Sandoz, Tokyo, Japan), and butorphanol (5 mg/kg; Meiji Seika Pharma Co., Ltd., Tokyo, Japan), and perfused through the heart with ice-cold phosphate-buffered saline (PBS; pH 7.4), immediately followed by a fixative containing 4% paraformaldehyde in PBS. Brain were then postfixed with the same fixative solution at 4 °C for 1 h and then placed in a 20% sucrose-buffered solution at 4 °C for 12 h. The brains were sliced into 40-µm sections from  -1.40 mm to -2.00 mm relative to bregma, using a cryostat (Cryostar NX70; Thermo Scientific, Waltham, MA, USA). Frozen sections were mounted on glass slides (Matsunami Glass, Osaka, Japan). After three 5-minute washes, the sections were incubated with PBS containing 1% normal goat serum and 0.3% Triton X-100 (PBSGT) at room temperature (23 ± 1 °C) for 2 h. They were then incubated overnight at 4 °C with a rabbit primary monoclonal antibody against ACE2 (1:100; Santa Cruz Biotechnology; Cat# sc-20,998), mouse anti-neuronal nuclear antigen antibody (NeuN; 1:200; Millipore, Burlington, USA; Cat# MAB377), mouse anti-glial fibrillary acidic protein antibody (GFAP; 1:200; Millipore; Cat# MAB360), and rabbit anti-ionized calcium binding adaptor molecule 1 antibodies (Iba-1; 1:200, Wako Pure Chemical Industries, Ltd., Osaka, Japan; Cat# 019–19741). After a 2-day incubation, the sections were washed twice with 0.1% PBS. When double labeling was performed using two primary antibodies from different host species (rabbit or mouse), sections were washed and incubated overnight at 4 °C with goat anti-rabbit IgG Alexa Fluor 568 (1:200; Molecular Probes, Eugene, USA; Cat# A11011) or goat anti-mouse IgG Alexa Fluor 488 (1:200; Molecular Probes; Cat# A11001) antibodies in PBSGT. When double labeling was performed using two primary antibodies from the same host species (rabbit anti-Iba-1 and rabbit anti-ACE2 antibodies), the detection of each antigen was performed sequentially and labeled goat anti-rabbit IgG Alexa Fluor 488 AffiniPure Fab fragment (1:80, Jackson ImmunoResearch Inc; Cat# 111-547-003), instead of whole antibodies, was used in the first detection (Iba-1). Thus, following the incubation with the rabbit anti-Iba-1 antibody, a goat monovalent Fab fragment tagged with fluorescent label 488 was added at a high concentration. This served two purposes: to label Iba-1 antibodies and to block/saturate their surface. The monovalent Fab fragment has only one antigen-binding site, which is used for binding to Iba-1, and therefore can not bind to the anti-ACE2 antibody used subsequently. The demonstration that the Fab fragment prevents binding of the secondary anti-rabbit Alexa 568 IgG (to detect the primary rabbit anti-ACE2 antibody on ACE2) to the primary anti-Iba-1 antibody is illustrated in Supplementary Fig. S3. In this control experiment, we show that there was no labeling by the anti-rabbit Alexa 568 IgG when immunostaining was performed without anti-ACE2 antibodies. The detailed Immunofluorescence staining methods for two primary antibodies from the same host species were carried out as previously described [43, 44]. DAPI (1:100; Wako Pure Chemical Industries) was used to stain and identify the nuclei. The sections were washed twice with 0.1% PBS and cover-slipped with fluorescent mounting medium (Dako, Carpinteria, CA, USA). The labeled sections were analyzed using a confocal laser scanning microscope (A1Rsi; Nikon, Tokyo, Japan).

### Statistical analysis

The experimental results are presented as mean ± standard error of the mean (SEM). The statistical significance of differences was determined by Student’s t*-*test for two-group comparisons. Significant differences were determined using one- or two-way analysis of variance (ANOVA), followed by the Tukey-Kramer test or Bonferroni test for multiple group comparisons. The criterion for a significant difference was set at p < 0.05.

## Electronic supplementary material

Below is the link to the electronic supplementary material.

Supplementary Material 1

## Data Availability

All data generated or analyzed during this study are included in this published article.

## Abbreviations

ACE: Angiotensin-converting-enzyme
Ang: Angiotensin
ANOVA: Analysis of variance
AGT: Angiotensinogen
AT1: Ang II Type 1
CA: Cornu ammonis
DG: Dentate gyrus
DIZE: Diminazene aceturate
GFAP: Glial fibrillary acidic protein
5-HT: Serotonin
i.c.v.: Intracerebroventricular
Iba-1: Ionized calcium binding adaptor molecule 1
NeuN: Neuronal nuclear antigen
PBS: Phosphate-buffered saline
PBSGT: PBS containing 1% normal goat serum and 0.3% Triton X-100
RAS: Renin-angiotensin system
SEM: Standard error of the mean
TBST: Tris-buffered saline supplemented with 0.01% Tween-20
