# Supplementary material for: Activation of angiotensin-converting enzyme 2 produces an antidepressant-like effect via MAS receptors in mice
Source: Mol Brain. 2023 Jun 13;16:52. doi: 10.1186/s13041-023-01040-y (PMC10262141; doi:10.1186/s13041-023-01040-y)
Supplement: Supplementary file 1 — Supplementary Material 1 [file 13041_2023_1040_MOESM1_ESM.docx]

**
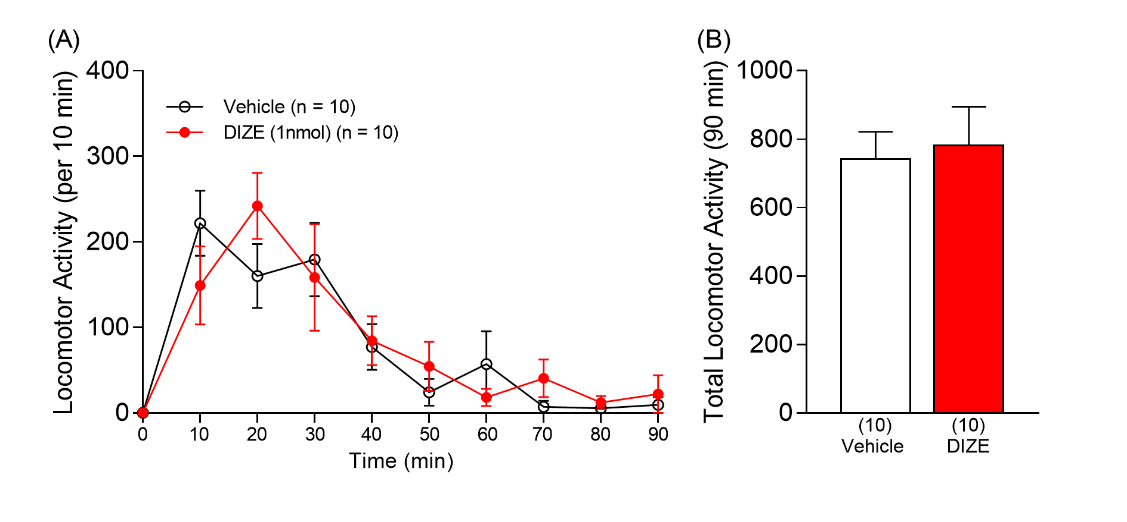
Supplemental information**

Fig. S1. Effects of DIZE on locomotor activity in mice. Time courses of locomotor activity (A) and total locomotor activity (B) were measured in mice between 0-90 min following the administration of vehicle or DIZE. Two-way ANOVA: [time: F (9, 180) = 14.13, p < 0.0001; treatment: F (1, 180) = 0.08793, p = 0.7672; time × treatment: F (9, 180) = 1.015, p = 0.4299, Fig. S1 (A)]. Student’s t-test: t = 0.2851, df = 18, p = 0.7788, Fig. S1 (B). Values represent the means ± SEM. Numbers in parentheses indicate the number of animals in each group.

**
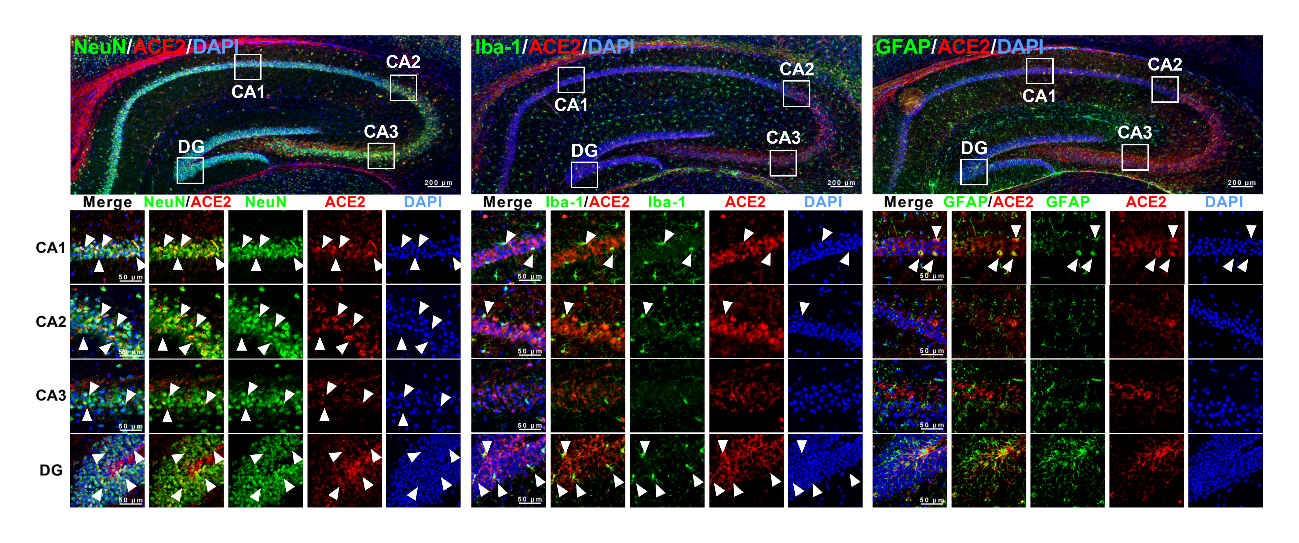
**

Fig. S2. Double immunofluorescence staining for ACE2 and cell specific markers for neuron (NeuN), microglia (Iba-1) or astrocyte (GFAP) in the hippocampus of naïve mice. Photomicrographs showing fluorescent labeling for ACE2 (red), NeuN (green), Iba-1 (green), GFAP (green) or nuclei with DAPI (blue) in the hippocampus. DG: dentate gyrus.


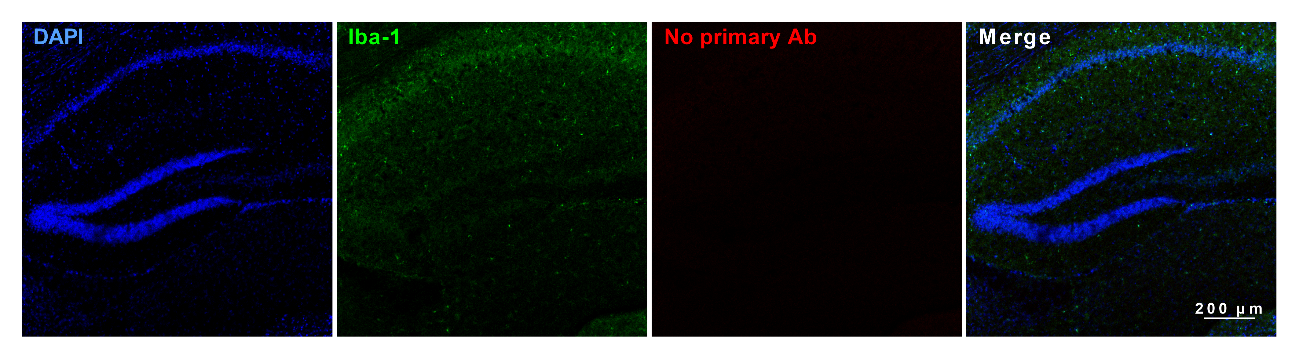
Fig. S3. Photomicrographs showing fluorescent labeling for Iba-1 (green), control for absence of Iba-1 labeling with second secondary antibody (Ab) (red), or nuclei with DAPI (blue), as well as merged images (right panels).
